# The Effect of Two Preservation Techniques on the Yield, Percentage Solids, Electrophoretic Profile, Gelatinolytic Activity, and Brine Shrimp Lethality of *Bitis arietans* Venom

**DOI:** 10.3390/molecules30183827

**Published:** 2025-09-21

**Authors:** Mitchel Okumu, Anna Nieczaj, Farhan Hassan, Selline Ooko, Ebrahim Sande, Rosa Chinheya, Jacqueline Manjia, Aleksandra Bocian

**Affiliations:** 1 Department of Public Health, Pharmacology and Toxicology, University of Nairobi, Nairobi P.O. Box 29053-00625, Kenya; farhanmohamud99@gmail.com; 2 Department of Research and Training, Jaramogi Oginga Odinga Teaching and Referral Hospital, Kisumu P.O. Box 849-40100, Kenya; 3 Department of Pharmacology, Faculty of Health Sciences, University of the Free State, Bloemfontein P.O. Box 339, South Africa; chinheya.r@ufs.ac.za (R.C.); manjianjikam.j@ufs.ac.za (J.M.); 4 Doctoral School, Rzeszow University of Technology, Al. Powstancow Warszawy 12, 35-959 Rzeszow, Poland; a.nieczaj@prz.edu.pl; 5 Department of Pure and Applied Chemistry, Masinde Muliro University of Science and Technology, Kakamega P.O. Box 190-50100, Kenya; sellineooko@mmust.ac.ke (S.O.); anyimba@gmail.com (E.S.); 6 Department of Biotechnology and Bioinformatics, Faculty of Chemistry, Rzeszow University of Technology, Al. Powstancow Warszawy 6, 35-959 Rzeszow, Poland

**Keywords:** *Bitis arietans* venom, freeze-drying, desiccator drying, mean venom yield, mean percentage solids, electrophoretic profile, gelatin in-gel zymography, brine shrimp lethality

## Abstract

This study compared the yield, percentage solids, electrophoretic profile, gelatinolytic activity, and brine shrimp lethality of *Bitis arietans* venom prepared using freeze-drying and desiccator drying. *Bitis arietans* venom was collected from snakes at Bioken snake farm, Kenya, whereafter it was pooled and divided into two parts. Part 1 was desiccator dried venom (DDV) while part 2 was freeze-dried venom (FDV). The yield and percentage solids in DDV and FDV were compared using Welch’s Student’s *t*-test and the dried venoms were subsequently subjected to sodium dodecyl sulphate polyacrylamide gel electrophoresis (SDS-PAGE), 2D electrophoresis, gelatin in-gel zymography, and brine shrimp lethality assays. Mean venom yield and percentage solids did not differ between DDV and FDV (*p* = 0.5647 and *p* = 0.4676, respectively). SDS-PAGE and two-dimensional (2D) electrophoresis revealed similar protein profiles for DDV and FDV, showing bands and spot clusters within molecular weight ranges of ~16 kDa to >150 kDa and pH ranging from 3.5 to 9.5. Enzyme zymography revealed comparable gelatinolytic activity between DDV and FDV. However, the brine shrimp lethality assay indicated significantly higher toxicity in DDV (LC_50_: 86.57 μg/mL) compared to FDV (LC_50_: 460.37 μg/mL). DDV also showed greater lethality than FDV at 100 μg/mL (*p* = 0.0416) and 1000 μg/mL (*p* = 0.0008) but not at 10 μg/mL (*p* = 0.2465). These findings suggest that DDV exhibits higher toxicity in brine shrimp larvae than FDV, although both drying methods result in similar yields, percentage solids, venom profile, and gelatinolytic activity. Further research is necessary to investigate the mechanism behind this difference and its implications for antivenom production and long-term stability of venom.

## 1. Introduction

Snake venoms are made up of biologically active heterogenous proteins and peptides tailored for defence, prey subjugation, and digestion [1,2]. The dominant protein families in snake venom include phospholipase A_2s_ (PLA_2s_), three finger toxins (3FTXs), and proteases, e.g., snake venom metalloproteases (SVMPs) or snake venom serine proteases (SVSPs). Less dominant proteins include Kunitz peptides (KUNs), cysteine-rich secretory proteins (CRISPs), L-amino acid oxidases (LAAOs), C-type lectins (CTLs), disintegrins (DIS), and natriuretic peptides (NP) [2,3,4].

Studies by Brunton, Fayrer, and Marsh on *Naja tripudians* and *Bitis gabonica* showed that liquid venom is unstable [5,6]. Previous research has also shown that the larger the protein, the more it adsorbs to a fixed surface, including in Lobind tubes which reduce, but do not eliminate, protein loss [7,8,9]. Munekiyo and Mackessy evaluated the stability of 15 aliquots of freshly extracted venom from the black-tailed rattlesnake *(Crotalus molossus molossus)* which was exposed to different storage temperatures (i.e., −80 °C to room temperature) [10]. The authors reported that a diluted sample of venom stored for one week at 37 °C was devoid of a number of high and low molecular weight bands relative to other venom samples [10]. Moreover, the authors observed an additional minor lower molecular weight band (~53 kDa) in the sample which was absent in all the other samples [10]. They posited that this band may have been the product of autolysis [10]. These observations underscore that proper preservation of snake venom is important to facilitate various applications including antivenom production, toxinological research, and drug discovery [10,11,12].

**Figure 1:**
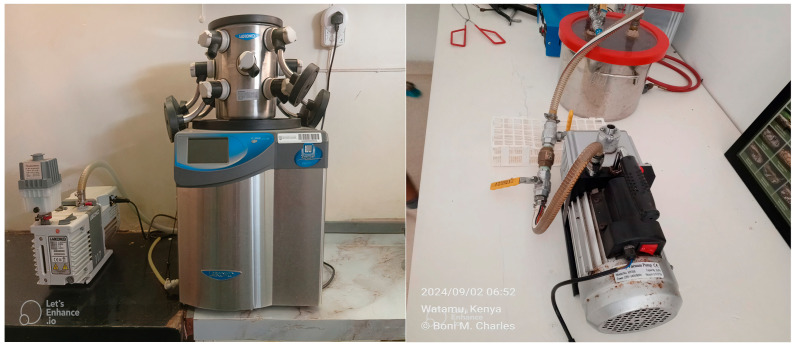
Image of a LabConco freeze-drier (LABCONCO Corporation, Kansas City, MO, USA) (**left**) maintained at the Department of Veterinary Anatomy and Physiology, University of Nairobi, Kenya Credit: Vivian Auma. Image of a rudimentary dessicator (Pegler Yorkshire Group Ltd., Doncaster, UK) (**right**) maintained at the Bioken snake farm in Watamu, Kenya. Credit: Charles Momanyi. The clarity of the images was sharpened using letsenhance.io.

Freeze-drying (lyophilization) is a process which dehydrates samples while they are frozen (Figure 1). It works on the principle of sublimation and through three steps namely freezing, primary drying, and secondary drying [13]. A freeze-dry apparatus primarily consists of a specimen chamber, a condenser chamber, and a vacuum pump [14]. Some freeze-driers may also have refrigeration components that maintain the dependability of the system [14].

The process of freeze drying confers proteins with a higher stability relative to the liquid form, particularly in the tropics where high temperatures may affect the integrity and activity of proteins (e.g., immunoglobulins) [15,16]. However, this technique has several limitations: too much heat in the process may alter the structure of the material, freezing damage may occur with labile products (e.g., proteins), conditions for conservative freeze-drying may result in a long processing time, and the process may lead to the production of unwanted eutectics [13,17,18]. The process also requires sophisticated capital-intensive equipment associated with high operation and energy (electricity) costs, which may not be readily available in many resource-limited snake venom collection facilities [14,19,20].

Desiccator drying is an alternative preservation method to freeze-drying. It requires minimal equipment and involves placing venom in a sealed container with a desiccating agent to remove moisture through passive dehydration [21,22]. In the system used at Bioken snake farm (Figure 1), silver beads are packed into an aluminum chamber to act as the desiccant. The beads are arranged in layers to maximize the surface area for moisture absorption, and approximately 300–500 g is used per drying cycle depending on venom volume. The chamber is then connected to a vacuum pump to create negative pressure, which accelerates dehydration by lowering the boiling point of water and drawing out moisture from the venom. Venom samples are placed in open containers within the chamber and left under these conditions until a constant dry weight is achieved, typically within 48–72 h. This low-cost modification of the conventional desiccator system enables effective preservation in resource-limited settings while avoiding the high energy and infrastructure demands of freeze-drying. Figure 1 below shows a LabConco freeze-drier (left) and a rudimentary desiccator (right) used to preserve pooled *Bitis arietans* venom samples in this study.

Whole snake venom is made up of water, macromolecules, and organic and inorganic compounds [23]. Venom yield is defined as the amount of venom produced by a snake when it bites. It is conventionally measured in its dry form after water has been eliminated [23]. The percentage of solids are the fraction of the solid weight of venom divided by the total weight of venom (solids and liquid) expressed as a percentage [(solid weight/total weight) × 100 = % Solids] [23].

SDS-PAGE is used to separate venom proteins based on their molecular weight [24]. Two-dimensional gel electrophoresis further enhances resolution of venom proteins by facilitating separation according to isoelectric point and size thereby facilitating detection of isoforms [24,25]. Gelatin in-gel zymography is an electrophoretic method that is useful in observing the activity of proteases including matrix metalloproteases (MMPs), metalloproteases, cysteine proteases, and serine proteases [26,27,28,29]. 

The brine shrimp (*Artemia salina*) lethality assay has emerged as a promising surrogate for murine dermonecrosis testing and offers a viable alternative that may reduce dependence on mice in snake venom research [30,31]. Because *Bitis arietans* is responsible for most of the bites in sub-Saharan Africa, and given that venom from this snake is known for exerting dermonecrosis, the aim of the present study was to compare the yield, percentage solids, protein profile, gelatinolytic (enzymatic) activity, and lethality (toxicity) of *Bitis arietans* venom prepared using either desiccator drying or freeze-drying.

## 2. Results

### 2.1. Yield and Percentage Solids in Venom

**Figure 2:**
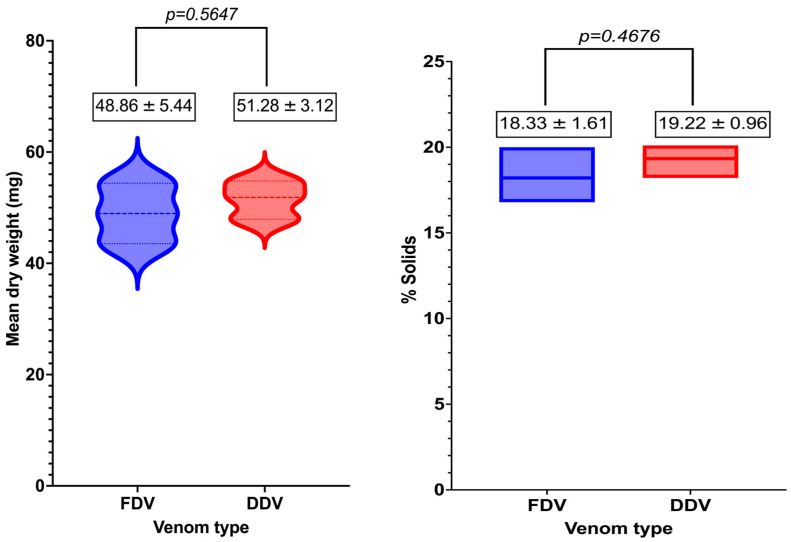
Violin plot of the mean dry weight of *Bitis arietans* venom prepared using freeze-drying (FDV) and desiccator drying (DDV) (**left**). Floating bars of the percentage of solids of *Bitis arietans* venom prepared using freeze-drying (FDV) and desiccator drying (DDV) (**right**).

Figure 2 shows the violin plot (left) and floating bars (right) comparing the mean dry weight and percentage of solids of *Bitis arietans* venom prepared using freeze-drying and desiccator drying. The mean dry weight of FDV (48.86 ± 5.44 mg) was not significantly different (*p* = 0.5647) from the mean dry weight of DDV (51.28 ± 3.12 mg).

Figure 2 shows the floating bars of the mean % solids in *Bitis arietans* venom prepared using freeze-drying and desiccator drying. The mean % solids in FDV (18.33 ± 1.61 mg) was not significantly different (*p* = 0.4676) from the mean % solids in DDV (19.22 ± 0.96 mg).

### 2.2. SDS-PAGE Electrophoresis

**Figure 3:**
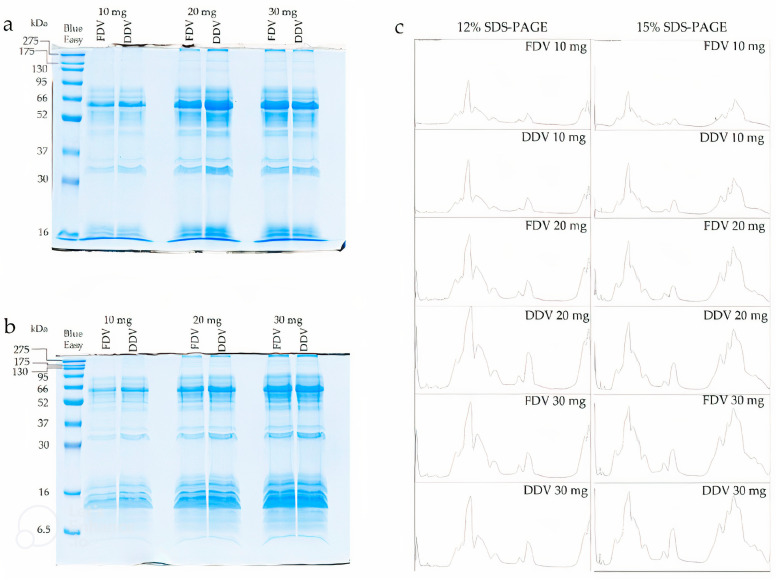
*Bitis arietans* venom analyses using SDS-PAGE electrophoresis. (**a**) 12% gel, (**b**) 15% gel, (**c**) plots for both gels made using ImageJ software. BlueEasy prestained protein marker with a mass range of 6.5–275 kDa was used for electrophoresis. The clarity of the images was sharpened using letsenhance.io.

SDS-PAGE electrophoresis was performed on two gel variants, namely 12% and 15%, and for three different amounts of protein applied to each well, specifically 10 mg, 20 mg, and 30 mg. Analysis using the SDS-PAGE technique revealed the presence of four main groups of proteins in the samples. The first, second, and third groups were proteins with masses above 150 kDa, around 60 kDa, and 35 kDa, respectively. The fourth group, however, were low-molecular-weight proteins with masses around 16 kDa. The exact protein profiles on the gels are as shown in Figure 3a,b and plots generated by ImageJ software (See Figure 3c). No qualitative differences were noted between FDV and DDV on the gels or in the plots. However, small quantitative differences were noted, as seen for the fraction around 35 kDa on the 12% gel (See Figure 3a).

### 2.3. 2D Electrophoresis

**Figure 4:**
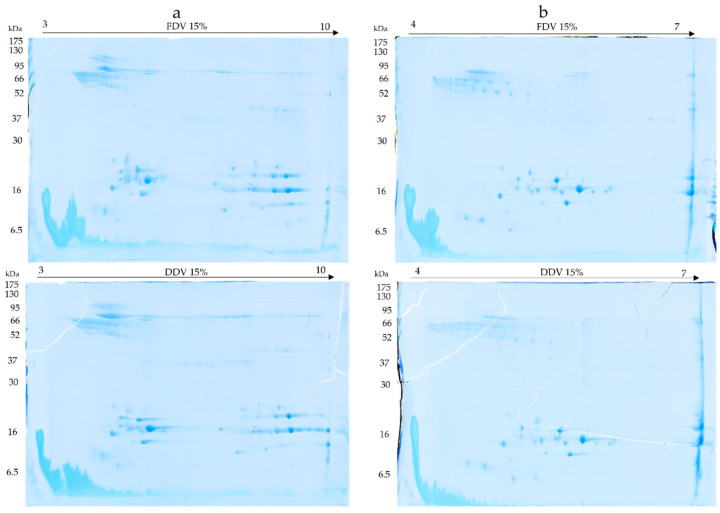
Gels generated by 2D electrophoresis technique using 15% gels. (**a**) Isoelectric focusing made on strips with a pH range of 3–10 for the FDV samples (**top**) and DDV samples (**bottom**), (**b**) Isoelectric focussing made on strips with a pH range of 4–7 for FDV samples (**top**) and DDV samples (**bottom**).

2D electrophoresis was performed in the first experiment on gel strips in the pH range 3–10 and 4–7 and SDS-PAGE gels with an acrylamide concentration of 15%. The gels with a pH range of 3–10 showed 3 large clusters of spots: pH 3.5–6 and masses of 52–130 kDa, pH 4.5–6 and 10–30 kDa, and pH 7–9.5 and 10–30 kDa (See Figure 4a). On the other hand, gels with a pH range of 4–7 showed two main clusters of spots: pH 4–5.5 and masses of 52–95 kDa, and pH 5–6 and masses of 10–30 KDa (see Figure 4b). No differences were found on the gels between FDV and DDV samples.

**Figure 5:**
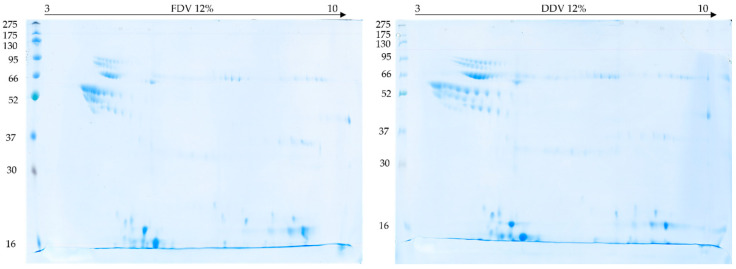
Two-dimensional gels of *Bitis arietans* venom samples FDV (**left**) and DDV (**right**) made using 3–10 Immobiline Dry Strip and 12% SDS-PAGE gels.

In the second experiment, gel strips with a pH range of 3–10 and 12% SDS-PAGE gels were used. This reduced the time of the second dimension (from 10 to 7 h) and better separated the high-molecular-weight proteins, but at the same time the low-molecular-weight proteins separated much more poorly (See Figure 5 below). Therefore, the gels do not show 3 distinct clusters of spots as on the gel in Figure 3, but nevertheless a group is visible in the pH range 3.5–5.5 and mass between 52 and 130 kDa, as well as spots at and above the border of the electrophoresis front in the pH ranges 4.5–6 and 7–9.5. Similarly, no differences between FDV and DDV samples were found on these gels either.

### 2.4. Gelatinolytic Activity Assay

**Figure 6:**
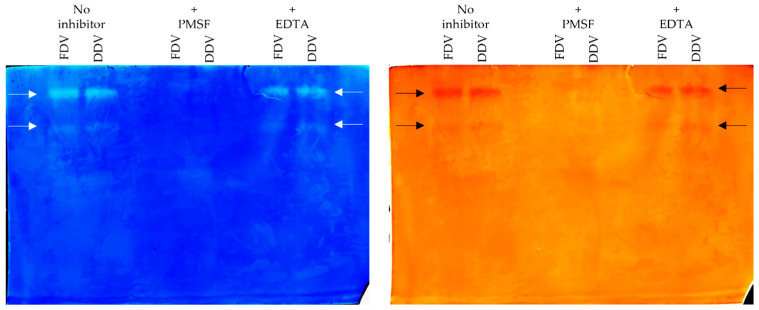
Zymography of *Bitis arietans* venom samples performed with porcine gelatin as a substrate. Samples were incubated with or without a protease inhibitor. Phenylmethylsulfonyl fluoride (PMSF) and ethylene diamine tetra acetic acid (EDTA) were used as protease inhibitors. The original gel after incubation in coomassie brilliant blue (CBB G250) dye is presented on the left and its negative on the right. The arrows show the bands that were digested in the gel.

Zymography was performed to examine the gelatinolytic activity of the venom samples. In both samples (FDV and DDV), two fractions were found to exhibit activity against gelatin, as evidenced by the two bands digested in the gel (See Figure 6 below). No activity was found in the two middle lanes when 1 mM Phenylmethylsulphonyl fluoride (PMSF) was used for incubation before electrophoresis, indicating that the observed enzymes belong to serine proteases. In contrast, the lack of effect of Ethylenediamine tetra acetic acid (EDTA) on the enzyme activity observed in the last two pathways on the right side of the gel indicates that the observed effect is not from metalloproteases. Likewise, no differences between FDV and DDV samples were found on these gels either.

### 2.5. Brine Shrimp Lethality Assay

Table 1 below shows the effect of exposing brine shrimp larvae to graded doses of DDV and FDV over 24 h. The concentration of DDV required to kill 50% of brine shrimp larvae was 86.57 µg/mL while the concentration of FDV required for a similar effect was 460.37 µg/mL.

**Figure 7:**
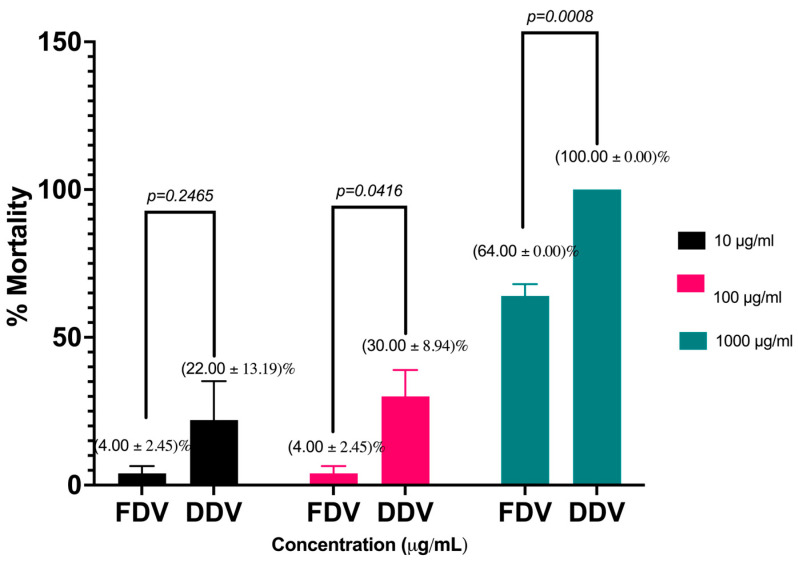
Student’s *t*-test comparison of the effect of graded FDV and DDV doses on brine shrimp larval mortality. Data expressed as Mean ± SEM and *p* < 0.05. DDV: Desiccator-dried venom, FDV: Freeze-dried venom, SEM: Standard Error of the Mean.

Figure 7 below represents a Student’s *t*-test comparison of the effect of graded doses of DDV and FDV on brine shrimp larvae. DDV-induced brine shrimp lethality was significantly higher than FDV-induced brine shrimp lethality at 100 μg/mL (*p* = 0.0416) and 1000 μg/mL (*p* = 0.0008) but not at 10 μg/mL (*p* = 0.2465).

## 3. Discussion

The present study is a comparison of two venom preservation techniques through analyses of venom yields, percentage solids, protein composition, enzymatic (gelatinolytic) activity, and toxicity of *Bitis arietans* venom. Our findings reveal both similarities and notable differences between freeze-dried (FDV) and desiccator-dried (DDV) venom samples.

Results on the comparison of the mean yield and % solids in *Bitis arietans* venom facilitated the acceptance of the null hypothesis that there is no difference in the mean yields and % solids in *Bitis arietans* venom prepared using either freeze-drying or desiccator drying. Moreover, the venom yield and % solids we have reported was comparable to what has been reported by previous workers [32,33]. The SDS-PAGE analysis revealed proteins across a broad molecular mass range (>150 kDa to ~16 kDa) in both DDV and FDV samples, suggesting that both preservation methods maintain the overall protein composition of *B. arietans* venom. The high molecular weight proteins (>150 kDa) associated with snake venom metalloproteinases (SVMPs), particularly P-III class metalloproteinases, are known to be abundant in viper venoms [34]. The proteins around 60 kDa may represent L-amino acid oxidases (LAAOs), which typically range between 57 and 70 kDa in snake venoms [35]. The bands around 35 kDa are consistent with snake venom serine proteases (SVSPs) and group II phospholipase A_2_ (PLA_2_) dimers, while the ~16 kDa bands likely represent PLA_2_ monomers and disintegrins [36].

The 2D electrophoresis revealed protein clusters across different pH ranges, providing additional information into the venom’s complexity. The large clusters observed at pH 3.5–6 (52–130 kDa) likely represent various isoforms of SVMPs and LAAOs, while the spots at pH 4–5.5 (52–95 kDa) may correspond to different glycoforms of these enzymes [3]. The clusters at pH 4.5–6 and pH 5–6 (10–30 kDa) are consistent with the typical distribution of SVSPs and PLA_2_s in viper venoms [37,38]. The basic proteins observed at pH 7–9.5 (10–30 kDa) may represent basic PLA_2_s and small basic peptides, which are common components in viper venoms [39,40,41]. The similarity in protein distribution patterns between DDV and FDV suggests that both preservation methods effectively maintain the primary structure of venom proteins. This observation may be of particular significance for resource-limited settings where freeze-drying equipment may not be available.

The presence of two distinct gelatinolytic (proteolytic) fractions in both samples may indicate that the studied methods preserve zinc-dependent catalytic domains of SVMPs essential for the observed gelatinolytic activity. This finding is particularly relevant for antivenom production, as SVMPs are not only important immunogens for generating therapeutic antibodies but they are also major contributors to the local tissue damage and haemorrhagic effects characteristic of *B. arietans* envenomation [42,43,44].

The most striking difference between the preservation methods was observed in the brine shrimp lethality assay, where DDV (LC_50_ = 86.57 µg/mL) showed significantly higher toxicity than FDV (LC_50_ = 460.37 µg/mL). This difference cannot be attributed to protein concentration, as all samples were equalized prior to analysis. A more plausible explanation is that during drying, venom proteins lose their native conformation and, depending on the preservation method, vary in their ability to regain structure upon rehydration. We hypothesize that the slower dehydration process in desiccator drying may better preserve the native conformation of certain toxins compared to the rapid freezing and sublimation involved in lyophilization which may partially denature temperature-sensitive toxins and reduce their activity. Although our analysis on the yield, percentage solids, electrophoretic, and enzymatic activity of DDV and FDV showed no differences, the marked divergence in toxicity between the two samples strongly suggests structural differences in toxin renaturation between methods. Regrettably, protein conformation analysis was beyond the scope of this study but represents an important avenue for future research. The current protocols for antivenom production and testing typically use freeze-dried venoms as reference standards [45]. Our findings suggest that this preservation method might significantly influence venom toxicity, potentially affecting the evaluation of antivenom efficacy.

How do these findings, in general, compare with similar studies in the literature? Schwick and Dickgiesser observed that the alkaline phosphatase activity of lyophilized crude *Dendroaspis polylepis* venom was nine times higher than the alkaline phosphatase activity of vacuum desiccated (silica gel/calcium chloride) venom. Conversely, the authors observed that the alkaline phosphatase activity in vacuum desiccated *Naja melanoleuca* venom was three times higher than the alkaline phosphatase activity in lyophilized crude venom. Unfortunately, the temperature and drying times for these experiments were not documented [46,47]. Another study by Munekiyo and Mackessy evaluated the effects of temperature and storage conditions on the electrophoretic, toxic, and enzymatic stability of *Crotalus molossus molossus* venom and reported that most venom activities remain stable even if the venom is stored or collected under potentially adverse conditions, and that freezing samples was not necessarily advantageous [10].

This study addresses an important gap in snake venom preservation by evaluating a resource-friendly alternative to freeze drying: a modified desiccator drying method which uses silver beads and negative pressure. These findings raise important questions about how preservation methods may influence venom bioactivity, with implications for antivenom production and stability, particularly in low-resource settings.

## 4. Materials and Methods

### 4.1. Collection and Preservation of Venom

*Bitis arietans* venom was collected from 10 wild-caught snakes, pooled, solubilized in 0.9% *w*/*v* saline solution, and centrifuged at 1000× *g* for 15 min. The diluted supernatant was aspirated through a 10 mL disposable syringe attached to a 0.45 μ membrane filter and divided into two portions (A and B). Portion A (800 mg wet weight) was transferred to three plastic containers and preserved using rudimentary desiccator equipment (Pegler Yorkshire Group Ltd., Doncaster, UK) maintained at the Bioken snake farm, Watamu, Kenya (DDV). Portion B was transferred to three plastic containers and lyophilized using a LabConco lyophilizer (LABCONCO Corporation, Kansas City, MO, USA) maintained at the Department of Veterinary Anatomy and Physiology, University of Nairobi, Kenya (FDV). All plastic containers (*n* = 6) were tared prior to venom transfer. Wet and dry weight of the venom were measured on a calibrated analytical balance and computed. The percentage solids were calculated according to the method described by Mirtschin and colleagues i.e., [23]

$$\mathrm{Percentage}\;\mathrm{solids}=\frac{Dry\;weight}{Wet\;weight}\;\times \;100$$

The difference in the dry mass (mg) and % solids between FDV and DDV were compared using Welch’s unpaired two-sided Student’s *t* test. Three independent observations were compared and results reported as mean ± SD with 95% Confidence Intervals on GraphPad Prism v 10.6.0 (796). *p* < 0.05 was considered significant.

### 4.2. SDS-PAGE Electrophoresis

Samples were prepared by dissolving 2 mg of dry venom in 1 mL of PBS buffer. SDS-PAGE electrophoresis was performed on 12% and 15% gels using a Mini-Protean Tetra cell apparatus (Bio-Rad Laboratories, Hercules, CA, USA) according to standard procedure [48]. Three series of samples containing 10 mg, 20 mg, and 30 mg of protein in 4× concentrated Laemmli buffer (375 mM Tris-HCl (pH 6.8), 9% SDS, 50% glycerol, 6% β-mercaptoethanol, 0.03% bromophenol blue) were prepared. BlueEasy Prestained Protein Marker (Nippon Genetics EUROPE, Duren, Germany) was used as the mass standard. After electrophoresis, the gels were stained overnight with colloidal Coomassie Brilliant Blue G-250 and then destained with deionized water [48]. Gels were scanned on an Image Scanner III (GE Healthcare, Chicago, IL, USA) and processed by ImageJ 1.52a software.

### 4.3. Two-Dimensional Electrophoresis

Samples were prepared by dissolving 2 mg of dry venom in 1 mL of deionized water. Two-Dimensional electrophoresis was performed according to the standard procedure described in detail earlier [25]. Briefly, the stock volume containing 550 µg of protein was diluted with urea-thiourea buffer (7 M urea, 2 M thiourea, 2% (*v*/*v*) Nonidet P-40, 0.5% (*v*/*v*) IPG buffer (pH range 3–10), 0.002% bromophenol blue, and 18 mM DTT) to a final volume of 450 µL [25]. Isoelectric focusing was performed on 24 cm long Immobiline DryStrip gels (GE Healthcare, Little Chalfont, UK) with a pH range of 4–7 and 3–10 on an Ettan™ IPGphor™ 3 IEF System (GE Healthcare, Little Chalfont, UK). The second dimension was carried out on 12% and 15% polyacrylamide gels on an Ettan DALTsix apparatus (GE Healthcare, Little Chalfont, UK) earlier Gels were stained and archived as described above.

### 4.4. Gelatinolytic Activity Assay

Enzyme zymography was performed according to the method described by Martinez [28] with some modifications on 10% SDS-PAGE gels containing 1.2% porcine gelatin. Stock volume (with a concentration of 2 mg/mL), containing 20 mg of protein dissolved in phosphate-buffered saline (PBS) was incubated for 30 min at room temperature without the addition of inhibitors and with the addition of 1 mM PMSF (serine protease inhibitor) and 1 mM EDTA (metalloprotease inhibitor) [28]. After incubation, 5 µL of 4× concentrated Laemmli buffer was added to the samples and a standard electrophoresis procedure was performed on a Mini-Protean Tetra cell apparatus (GE Healthcare, Little Chalfont, UK). After separation, the gels were washed three times each in 2.5% triton X-100 for 15 min, incubated overnight at 37 °C in 50 mM phosphate buffer pH 6.8 with 5 mM L-cysteine and 0.1% triton X-100, and stained with colloidal Coomassie Brilliant Blue G-250 (GE Healthcare, Little Chalfont, UK) the next day [28].

### 4.5. Brine Shrimp Lethality Assay

The method described by Meyer and colleagues was used [49] with modifications as described previously [30,50]. Briefly, graded doses (10 μg/mL, 100 μg/mL, and 1000 μg/mL) of DDV and FDV were tested in brine shrimp larvae (*Artemia salina*) using 5 mL plastic sample vials. Five tubes were used per test dose, and each tube contained 10 animals. The mortality of brine shrimp larvae was evaluated after 24 h. Probit regression analysis on the Statistical Package for the Social Sciences (SPSS, v 25.0) was used to calculate the concentration of DDV and FDV which killed 50% of the brine shrimp. Differences in the mortality induced by DDV and FDV were evaluated on GraphPad Prism Version 10.6.0 (796), expressed as Mean ± SEM, tested for normality using the Shapiro–Wilk test, and analysed using Welch’s unpaired two-tailed *t*-test. The confidence level was set at 95% and *p* < 0.05 was considered significant.

### 4.6. Ethical Considerations

Ethical approval to conduct this study was obtained from the Biosafety, Animal Use, and Ethics Committee of the Faculty of Veterinary Medicine, University of Nairobi (REF FVM BAUEC/2019/220).

## 5. Conclusions

These findings suggest that desiccator drying and freeze-drying of *Bitis arietans* venom produces similar venom yield, percentage solids, and protein profiles but different toxicity levels. The higher toxicity of DDV warrants investigation into whether dessicator drying may be advantageous over freeze drying for certain research applications, particularly those focusing on venom toxins in their most active forms. It may also be important to investigate the structural basis for the observed differences in toxicity. 

## Figures and Tables

**Table 1:** Brine shrimp lethality of *Bitis arietans* venom prepared using different preservation techniques.

Venom Type	Concentration (µg/mL)	Number of Deaths per 5 mL Sample Tube (Out of 10 Larvae)					Mean % Number of Deaths ± SEM	LC_50_ (µg/mL)	Toxicity	
		I	II	III	IV	V			Meyer’s Toxicity Index	Clarkson’s Toxicity Index
DDV	10 100 1000	1 5 10	3 4 10	7 0 10	0 4 10	0 2 10	22.00 ± 13.19 30.00 ± 8.94 100 ± 0.00	86.57	Toxic	Highly cytotoxic
FDV	10 100 1000	0 0 6	1 1 5	0 1 7	1 0 7	0 0 7	4.00 ± 2.45 4.00 ± 2.45 64.00 ± 4.00	460.37	Toxic	Slightly (low) cytotoxic

LC_50_: Lethal concentration required to kill 50% of brine shrimp larvae; DDV: Desiccator-dried venom, FDV: Freeze-dried venom, SEM: Standard Error of the Mean, µg/mL: microgram per milliliter.

## Data Availability

The data presented in this study are available on reasonable request from the corresponding author.

## Abbreviations

The following abbreviations are used in this manuscript:

DDV	Desiccator dried venom
FDV	Freeze-dried venom
2D	Two-dimensional
SDS-PAGE	Sodium Dodecyl Sulphate-Polyacrylamide Gel Electrophoresis
KDa	Kilodaltons
LC_50_	Lethal concentration responsible for the death of 50% of brine shrimp
PLA_2s_	Phospholipase A2’s
3FTXs	Three Finger Toxins
SVMPs	Snake Venom Metalloproteases
SVSPs	Snake Venom Serine Proteases
KUN	Kunitz Peptides
CRISP	Cysteine Rich Secretory Protein
LAAO	L-Amino Acid Oxidase
CTL	C-type Lectins
DIS	Disintegrins
NP	Natriuretic peptide
IEF	Isoelectric Focusing
EDTA	Ethylene Diamine Tetra Acetic Acid
PMSF	Phenylmethylsulphonyl fluoride
CBB	Coomassie Brilliant Blue
SPSS	Statistical Package for the Social Sciences
FVM	Faculty of Veterinary Medicine
BAUEC	Biosafety, Animal Use, and Ethics Committee

## References

[1] Rao S., Reghu N., Nair B.G., Vanuopadath M. (2024). The Role of Snake Venom Proteins in Inducing Inflammation Post-Envenomation: An Overview on Mechanistic Insights and Treatment Strategies. Toxins.

[2] Tasoulis T., Isbister G.K. (2023). A Current Perspective on Snake Venom Composition and Constituent Protein Families. Arch. Toxicol..

[3] Tasoulis T., Isbister G.K. (2017). A Review and Database of Snake Venom Proteomes. Toxins.

[4] Tasoulis T., Pukala T.L., Isbister G.K. (2022). Investigating Toxin Diversity and Abundance in Snake Venom Proteomes. Front Pharmacol..

[5] Willemse G.T., Hattingh J. (1978). Snake Venom Instability. Afr. Zool.

[6] Brunton T.L., Fayrer J. (1875). On the Nature and Physiological Action of the Poison of Naja Tripudians and Other Indian Venomous Snakes. Ind. Med. Gaz..

[7] Rabe M., Verdes D., Seeger S. (2011). Understanding Protein Adsorption Phenomena at Solid Surfaces. Adv. Colloid Interface Sci..

[8] Vogler E.A. (2012). Protein Adsorption in Three Dimensions. Biomaterials.

[9] Grzeskowiak R., Hamels S., Gancarek E. (2016). Comparative Analysis of Protein Recovery Rates in Eppendorf LoBind^®^ and Other “Low Binding” Tubes.

[10] Munekiyo S.M., Mackessy S.P. (1998). Effects of Temperature and Storage Conditions on the Electrophoretic, Toxic and Enzymatic Stability of Venom Components. Comp. Biochem. Physiol. B Biochem. Mol. Biol..

[11] Jesupret C., Baumann K., Jackson T.N.W., Ali S.A., Yang D.C., Greisman L., Kern L., Steuten J., Jouiaei M., Casewell N.R. (2014). Vintage Venoms: Proteomic and Pharmacological Stability of Snake Venoms Stored for up to Eight Decades. J. Proteom..

[12] Almeida J.R., Mendes B., Patiño R.S.P., Pico J., Laines J., Terán M., Mogollón N.G.S., Zaruma-Torres F., Caldeira C.A.D.S., da Silva S.L. (2020). Assessing the Stability of Historical and Desiccated Snake Venoms from a Medically Important Ecuadorian Collection. Comp. Biochem. Physiol. Part C Toxicol. Pharmacol..

[13] Shukla S. (2011). Freeze Drying Process: A Review. Int. J. Pharm. Sci. Res..

[14] Hower R.O. (1967). The Freeze-Dry Preservation of Biological Specimens.

[15] Santos L., Oliveira C., Vasconcelos B.M., Vilela D., Melo L., Ambrósio L., da Silva A., Murback L., Kurissio J., Cavalcante J. (2021). Good Management Practices of Venomous Snakes in Captivity to Produce Biological Venom-Based Medicines: Achieving Replicability and Contributing to Pharmaceutical Industry. J. Toxicol. Environ. Health Part B.

[16] Herrera M., Solano D., Gómez A., Villalta M., Vargas M., Sánchez A., Gutiérrez J.M., León G. (2017). Physicochemical Characterization of Commercial Freeze-Dried Snake Antivenoms. Toxicon.

[17] Brülls M., Rasmuson A. (2002). Heat Transfer in Vial Lyophilization. Int. J. Pharm..

[18] Wei W., Mo C., Guohua C. (2012). Issues in Freeze Drying of Aqueous Solutions. Chin. J. Chem. Eng..

[19] Patra A., Herrera M., Gutiérrez J.M., Mukherjee A.K. (2021). The Application of Laboratory-Based Analytical Tools and Techniques for the Quality Assessment and Improvement of Commercial Antivenoms Used in the Treatment of Snakebite Envenomation. Drug Test Anal..

[20] Dalhat M.M., Potet J., Mohammed A., Chotun N., Tesfahunei H.A., Habib A.G. (2023). Availability, Accessibility and Use of Antivenom for Snakebite Envenomation in Africa with Proposed Strategies to Overcome the Limitations. Toxicon X.

[21] Dorce V.A.C., da Rocha M.M.T., Candido D.M., Nencioni A.L.A., Auada A.V.V., Barbaro K.C., Lebrun I. (2018). Influence of Different Processing Techniques on the Toxicity and Biochemical Characteristics of Tityus Serrulatus Scorpion Venom. Toxicon.

[22] Egen N.B., Russell F.E. (1984). Effects of Preparatory Procedures on the Venom from a Rattlesnake (Crotalus Molossus Molossus), as Determined by Isoelectric Focusing. Toxicon.

[23] Mirtschin P.J., Shine R., Nias T.J., Dunstan N.L., Hough B.J., Mirtschin M. (2002). Influences on Venom Yield in Australian Tigersnakes (*Notechis scutatus*) and Brownsnakes (*Pseudonaja textilis*: *Elapidae*, *Serpentes*). Toxicon.

[24] Sahyoun C., Rima M., Mattei C., Sabatier J.-M., Fajloun Z., Legros C. (2022). Separation and Analytical Techniques Used in Snake Venomics: A Review Article. Processes.

[25] Hus K.K., Buczkowicz J., Pietrowska M., Petrilla V., Petrillová M., Legáth J., Litschka-Koen T., Bocian A. (2024). Venom Diversity in Naja Mossambica: Insights from Proteomic and Immunochemical Analyses Reveal Intraspecific Differences. PLoS Negl. Trop. Dis..

[26] Every D. (1981). Quantitative Measurement of Protease Activities in Slab Polyacrylamide Gel Electrophoretograms. Anal. Biochem..

[27] Vandooren J., Geurts N., Martens E., Van den Steen P.E., Opdenakker G. (2013). Zymography Methods for Visualizing Hydrolytic Enzymes. Nat. Methods.

[28] Meléndez-Martínez D., Plenge-Tellechea L.F., Gatica-Colima A., Cruz-Pérez M.S., Aguilar-Yáñez J.M., Licona-Cassani C. (2020). Functional Mining of the Crotalus Spp. Venom Protease Repertoire Reveals Potential for Chronic Wound Therapeutics. Molecules.

[29] Kleiner D.E., Stetlerstevenson W.G. (1994). Quantitative Zymography: Detection of Picogram Quantities of Gelatinases. Anal. Biochem..

[30] Okumu M.O., Mbaria J.M., Gikunju J.K., Mbuthia P.G., Madadi V.O., Ochola F.O., Jepkorir M.S. (2021). Artemia Salina as an Animal Model for the Preliminary Evaluation of Snake Venom-Induced Toxicity. Toxicon X.

[31] Araya X., Okumu M., Durán G., Gómez A., Gutiérrez J.M., León G. (2024). Assessment of the Artemia Salina Toxicity Assay as a Substitute of the Mouse Lethality Assay in the Determination of Venom-Induced Toxicity and Preclinical Efficacy of Antivenom. Toxicon X.

[32] Mallow D., Ludwig D., Nilson G. (2003). True Vipers: Natural History and Toxinology of Old-World Vipers.

[33] Brown J.H. (1973). Toxicology and Pharmacology of Venoms from Poisonous Snakes.

[34] Fox J.W., Serrano S.M.T. (2008). Exploring Snake Venom Proteomes: Multifaceted Analyses for Complex Toxin Mixtures. Proteomics.

[35] Du X.-Y., Clemetson K.J. (2002). Snake Venom L-Amino Acid Oxidases. Toxicon.

[36] Eichberg S., Sanz L., Calvete J.J., Pla D. (2015). Constructing Comprehensive Venom Proteome Reference Maps for Integrative Venomics. Expert Rev. Proteom..

[37] Ghezellou P., Albuquerque W., Garikapati V., Casewell N.R., Kazemi S.M., Ghassempour A., Spengler B. (2020). Integrating Top-down and Bottom-up Mass Spectrometric Strategies for Proteomic Profiling of Iranian Saw-Scaled Viper, Echis Carinatus Sochureki, Venom. J. Proteome Res..

[38] Offor B.C., Muller B., Piater L.A. (2022). A Review of the Proteomic Profiling of African Viperidae and Elapidae Snake Venoms and Their Antivenom Neutralisation. Toxins.

[39] Fernandes C.A.H., Borges R.J., Lomonte B., Fontes M.R.M. (2014). A Structure-Based Proposal for a Comprehensive Myotoxic Mechanism of Phospholipase A2-like Proteins from Viperid Snake Venoms. Biochim. Biophys. Acta (BBA)—Proteins Proteom..

[40] Gasanov S.E., Dagda R.K., Rael E.D. (2014). Snake Venom Cytotoxins, Phospholipase A2s, and Zn^2+^-Dependent Metalloproteinases: Mechanisms of Action and Pharmacological Relevance. J. Clin. Toxicol..

[41] de Oliveira A.L.N., Lacerda M.T., Ramos M.J., Fernandes P.A. (2024). Viper Venom Phospholipase A2 Database: The Structural and Functional Anatomy of a Primary Toxin in Envenomation. Toxins.

[42] Gutiérrez J.M., Rucavado A., Escalante T. (2016). Snake Venom Metalloproteinases. Handbook of Venoms and Toxins of Reptiles.

[43] Bermúdez-Méndez E., Fuglsang-Madsen A., Føns S., Lomonte B., Gutiérrez J.M., Laustsen A.H. (2018). Innovative Immunization Strategies for Antivenom Development. Toxins.

[44] Alomran N., Alsolaiss J., Albulescu L.-O., Crittenden E., Harrison R.A., Ainsworth S., Casewell N.R. (2021). Pathology-Specific Experimental Antivenoms for Haemotoxic Snakebite: The Impact of Immunogen Diversity on the in Vitro Cross-Reactivity and in Vivo Neutralisation of Geographically Diverse Snake Venoms. PLoS Negl. Trop. Dis..

[45] WHO (2018). Guidelines for the Production, Control and Regulation of Snake Antivenom Immunoglobulins.

[46] Fix J.D. (1976). Short Communication Cryosorptive Pumping as a Method of Lyophilization and Vacuum Desiccation of Snake Venom.

[47] Schwick G., Dickgiesser F. (1963). Probleme Der Antigen-Und Fermentanalyse Im Zusammenhang Mit Der Herstellung Polyvalenter Schlangengiftseren. Gift. Erde.

[48] Laemmli U.K. (1970). Cleavage of Structural Proteins during the Assembly of the Head of Bacteriophage T4. Nature.

[49] Meyer B.N., Ferrigni N.R., Putnam J.E., Jacobsen L.B., Nichols D.E.J., McLaughlin J.L. (1982). Brine Shrimp: A Convenient General Bioassay for Active Plant Constituents. Planta Med..

[50] Okumu M., Mbaria J., Gikunju J., Mbuthia P., Madadi V., Ochola F., Oketch-Rabah H., Dhanani T., Kalita B., Vishwa Vidyapeetham A. (2024). Exploring Nature’s Antidote: Unveiling the Inhibitory Potential of Selected Medicinal Plants from Kisumu, Kenya against Venom from Some Snakes of Medical Significance in Sub-Saharan Africa. Front. Pharmacol..

